# Proceedings of resources for optimal care of acute care and emergency surgery consensus summit Donegal Ireland

**DOI:** 10.1186/s13017-017-0158-x

**Published:** 2017-10-23

**Authors:** M. Sugrue, R. Maier, E. E. Moore, M. Boermeester, F. Catena, F. Coccolini, A. Leppaniemi, A. Peitzman, G. Velmahos, L. Ansaloni, F. Abu-Zidan, P. Balfe, C. Bendinelli, W. Biffl, M. Bowyer, M. DeMoya, J. De Waele, S. Di Saverio, A. Drake, G. P. Fraga, A. Hallal, C. Henry, T. Hodgetts, L. Hsee, S. Huddart, A. W. Kirkpatrick, Y. Kluger, L. Lawler, M. A. Malangoni, M. Malbrain, P. MacMahon, K. Mealy, M. O’Kane, P. Loughlin, M. Paduraru, L. Pearce, B. M. Pereira, A. Priyantha, M. Sartelli, K. Soreide, C. Steele, S. Thomas, J. L. Vincent, L. Woods

**Affiliations:** 1 Department of Surgery, Letterkenny University Hospital and Donegal Clinical Research Academy, Donegal, Ireland; 2 Department of Surgery, University of Washington, Seattle, USA; 3 Harborview Medical Center, Seattle, USA; 4 University of Colorado, Denver, USA; 5 Department of Surgery, Academic Medical Center, Amsterdam, Netherlands; 6 Department of Emergency Surgery, Maggiore Hospital, Parma, Italy; 7 Department of Emergency, General and Transplant Surgery, Papa Giovanni XXIII Hospital, Bergamo, Italy; 8 Abdominal Center, University Hospital Meilahti, Helsinki, Finland; 9 Department of Surgery, University of Pittsburgh School of Medicine, Pittsburgh, PA USA; 10 Department of Trauma, Emergency Surgery and Surgical Critical Care, Massachusetts General Hospital, Boston, MA USA; 11 General Surgery Department, Papa Giovanni XXIII Hospital, Bergamo, Italy; 12 Department of Surgery, College of Medicine and Health Sciences, UAE University, Al-Ain, United Arab Emirates; 13 Department of Surgery, St. Luke’s Hospital, Kilkenny, Ireland; 14 Department of Surgery, John Hunter Hospital, Newcastle, NSW Australia; 15 Acute Care Surgery, The Queens Medical Center, Honolulu, HI USA; 16 Department of Surgery, Uniformed Services University and Walter Reed National Military Medical Center, Bethesda, MD USA; 17 Department of Trauma/Critical Care, Massachusetts General Hospital, Boston, MA USA; 18 Department of Critical Care Medicine, Ghent University Hospital, Ghent, Belgium; 19 Maggiore Hospital of Bologna, AUSL, Bologna, Italy; 20 Letterkenny University Hospital and Donegal Clinical Research Academy, Donegal, Ireland; 21 Division of Trauma Surgery, Department of Surgery, School of Medical Sciences, University of Campinas, Campinas, Brazil; 22 Department of Surgery, American University of Beirut Medical Center, Beirut, Lebanon; 23 National Clinical Advisor for the Acute Hospitals Division, Health Service Executive, Dublin, Ireland; 24 Trauma Governance, UK Defence Medical Services, Lichfield, UK; 25 Department of Trauma and Acute Care Surgery, Auckland City Hospital, Auckland, New Zealand; 26 Department of Anaesthesiology, Royal Surrey County Hospital, Guildford, UK; 27 Department of Surgery, Critical Care Medicine and Regional Trauma Service, Foothills Medical Centre, Calgary, AB Canada; 28 Department of General Surgery, Division of Surgery, Rambam Health Care Campus, Haifa, Israel; 29 Department of Radiology, Mater Misericordiae University Hospital, Dublin, Ireland; 30 American Board of Surgery, Philadelphia, PA USA; 31 Intensive Care Unit and High Burn Unit, ZNA “Ziekenhuis Netwerk Antwerpen” Stuivenberg and ZNA St-Erasmus hospitals, Antwerp, Belgium; 32 Department of Radiology, Mater Misericordiae University Hospital, Dublin, Ireland; 33 Department of Surgery, Wexford University Hospital, Wexford, Ireland; 34 Department of Pathology, Altnagelvin Hospital, Londonderry, UK; 35 Department of Surgery, Altnagelvin Hospital, Londonderry, UK; 36 Department of General and Emergency Surgery, Milton Keys, UK; 37 Northwest Research Collaborative, Manchester, UK; 38 Division of Trauma Surgery, Department of Surgery, School of Medical Sciences, University of Campinas, Campinas, Brazil; 39 Department of Gastroenterology, Teaching Hospital, South, Colombo, Sri Lanka; 40 Department of Surgery, Macerata Hospital, Macerata, Italy; 41 Department of Clinical Medicine, University of Bergen, Bergen, Norway; 42 Department of Gastroenterology, Letterkenny University Hospital and Donegal Clinical Research Academy, Donegal, Ireland; 43 Department of Trauma Services, Memorial Hospital of South Bend, Indiana, USA; 44 Department of Intensive Care, Erasme Hospital, Université libre de bruxelles, Brussels, Belgium; 45 Department of Acute Hospitals, Health Services Executive, Dublin, Ireland; 46 Department of Gastrointestinal Surgery, Stavanger University Hospital, Stavanger, Norway

**Keywords:** Emergency surgery, Optimal care, Performance indicators, Surgical outcomes

## Abstract

**Background:**

Opportunities to improve emergency surgery outcomes exist through guided better practice and reduced variability. Few attempts have been made to define optimal care in emergency surgery, and few clinically derived key performance indicators (KPIs) have been published. A summit was therefore convened to look at resources for optimal care of emergency surgery. The aim of the Donegal Summit was to set a platform in place to develop guidelines and KPIs in emergency surgery.

**Methods:**

The project had multidisciplinary global involvement in producing consensus statements regarding emergency surgery care in key areas, and to assess feasibility of producing KPIs that could be used to monitor process and outcome of care in the future.

**Results:**

Forty-four key opinion leaders in emergency surgery, across 7 disciplines from 17 countries, composed evidence-based position papers on 14 key areas of emergency surgery and 112 KPIs in 20 acute conditions or emergency systems.

**Conclusions:**

The summit was successful in achieving position papers and KPIs in emergency surgery. While position papers were limited by non-graded evidence and non-validated KPIs, the process set a foundation for the future advancement of emergency surgery.

## Background

Optimal consistent emergency surgery care presents a major health challenge worldwide [1–3]. Patients requiring urgent surgical care are often critically ill with significant pre-existing comorbidities [4]. While there is a wide spectrum of potential presenting surgical conditions, there is a predictable pattern because the top seven emergency surgery conditions account for nearly 80% of presentations [5]. Modern surgical care requires a multi-disciplinary approach and streamlined acute pathways are critical to ensure optimal outcomes [6].

Historically, it is not uncommon to manage emergency surgical patients interspersed with daily elective activities within a given hospital system [7]. The lack of timely appropriate access to emergency surgical care is often multi-factorial and may include shortage of emergency surgeons, inadequate access to the operating room, lack of a dedicated team, and a paucity of clinical pathways [8].

Over the past decade, the importance of a comprehensive system in managing emergency surgical care has become evident, resulting in training bodies and health ministries publishing multiple consensus papers and statements on this topic [6, 9–13].

Monitoring emergency surgery performance and outcomes is essential and clinicians themselves need to be involved in determining key performance indicators (KPIs). KPIs in emergency surgery have not been widely developed. For this reason, under the leadership of the World Society of Emergency Surgery, with support from the Abdominal Compartment Society and Donegal Clinical Research Academy key opinion leaders in the field of emergency surgery care across many disciplines were invited to contribute to a Performance Summit in Donegal in 2016.

The Emergency Surgery Performance Summit aimed to develop key performance indicators in clinical and systems delivery that would lay the foundation for future optimal surgery development.

## Methods

Common aspects of emergency surgery were identified into 14 categories (Table 1), 44 key opinion leaders were invited to participate and co-author individual chapters. There were 14 position papers and 20 topics for KPI development (Table 2). A review of published articles and consensus statements relating to the establishment and design of emergency, acute care surgery, and emergency general services was performed. Emergency surgery position statements from the surgical colleges, surgical institutions and key government organisations were assessed. The key performance indicators were proposed according to a standardised pro forma (Table 3). Each KPI had to be easily measured and reproducible. Due to the extent and complexity of topics and number of authors, the original intent to grade level was not uniform and thus reporting was confined to consensus opinion.

**Table 1:** Key position topics for summit

Resources and designation of emergency surgery	
Acute care unit structure	
Reception and triage	
Data systems, registry and evaluation	
Rural emergency care and transfer	
Paediatric emergency care	
Geriatric emergency care	
Interaction and laboratory, radiology, ICU gastroenterology	
Quality assurance and performance improvement	
Sepsis control in emergency room	
Research in acute care surgery	
Education in emergency surgery	
Accreditation review and consultative program	
Patient related outcomes measures

**Table 2:** Key performance indicators topics

Appendicitis	
Cholecystitis	
Pancreatitis	
Perforated ulcer	
Gastrointestinal bleeding	
Bowel obstruction	
Diverticulitis	
Mesenteric ischaemia	
Abdominal vascular emergencies	
Coagulation	
Complex pneumothorax and empyema	
Septic shock in emergency; ICU	
Fluid resuscitation in septic shock	
Abdominal compartment syndrome	
Geriatric care	
Triage; ICU admission	
Laboratory	
Wound care	
Emergency theatre	
Health care systems

**Table 3:** Example of KPI of 1 of the 112 KPI generated

Title	Negative appendectomy rate
Description	Percentage of negative appendectomies performed
Rationale	It is an indicator of diagnostic efficiency. In order to avoid unnecessary surgery and decrease costs and complications.
Target	< 10% appendixes removed are normal
KPI collection frequency	Annually
KPI reporting frequency	Annually
KPI calculation	Numerator divided by denominator expressed as a percentage Numerator: number of patients underwent appendectomy with negative appendectomy Denominator: number of all patients underwent appendectomy
Reporting aggregation	Hospital, hospital group
Data source(s)	OR registry, medical records, patients chart, hospital discharge data, emergency surgery database

## Results

The summit was held in Lough Eske Castle Donegal Ireland on 25 July 2016, attended by 80 people of which 44 contributed to writing the Proceeding’s chapters, and associated KPIs. The key opinion leaders were from seven disciplines, predominantly surgery, but also including critical care, internal medicine, emergency medicine, radiology and nursing. There were 119 KPIs described for the 20 conditions, a sample is shown in Table 3. The entire proceedings for the summit are available on line [14]. The summit provided a platform for discussion and agreed consensus on the key position topics. Future resources for advancing systems, clinical care, research and reporting were debated and supported. Consensus was reached that the KPIs for use in emergency surgery care needed to be simple, with a small number for each major condition.

## Discussion

Globally, there is increasing interest in improving emergency surgery outcomes by health providers, learned societies, colleges and health departments [15–17]. Over a decade ago, it was estimated that more than 230 million surgical procedures were performed and within that workload, emergency general surgery accounts for a significant part [18]. In addition, emergency surgery has one of the greatest overall associated mortalities of any medical discipline [19]. It is estimated that 890,000 patients die during their emergency surgical care annually [20]. Patients undergoing laparotomy have variable mortality depending on their diagnosis, treatment and location of service provision [1, 2, 4, 21]. The American College of Surgeons National Surgical Quality Improvement Program database identified that emergency surgery patients have significantly more postoperative complications (23 vs 14%; *P* < .0001) as well as greater mortality rates (6 vs 1%; *P* < .0001) compared with non-emergency general surgery patients [22]. Ingraham recently reported that an expert panel ranked quality indicators in certain emergency surgery conditions [23]. They reviewed historic compliance with select quality indicators for four procedures (cholecystectomy, appendectomy, colectomy, small bowel resection) at four academic centres and concluded that potential adherence to quality indicators may improve the quality of emergency general surgery care provided for which current outcomes are potentially modifiable [23]. The summit reported KPIs in a much larger group, incorporating 20 conditions and sectors of health care provision.

To improve outcomes, we must not just develop quality benchmarks and standards but also understand prevalence and significance of complications [24, 25]. While there are limitations to many new systems being developed [26, 27]. It is only through engagement with all the disciplines involved in emergency surgery that care will evolve and improve. The Donegal Summit on resources for optimal care included not just surgeons, but also emergency physicians, anaesthetists, critical care, internal medicine, gastroenterology, radiology and nursing. While the summit developed and reported potential key performance indicators and outlines of basic resources required for functioning part of emergency surgery systems, it had limitations. There was inadequate patient forum representation. The process was consensus-based and did not use a formal statistical or Delphi approach for the development of KPIs. The KPIs would in time need to be validated.

The summit and this proceedings paper have however set a process in place to facilitate concepts and benchmarks in resourcing emergency surgery. It has mirrored that international desire to improve outcomes [24].

Over the last decade there has been increasing development of Acute Care Surgical Units. Some of these have developed and reported limited KPIs [7]. Trauma care has been to the forefront of KPI development in acute care. In other areas of surgery, KPIs are widely reported. This summit was unique in having many key opinion leaders in attendance and discussing the process.

## Conclusion

In conclusion, the Summit on Resources for Optimal Care of Acute Care and Emergency Surgery Consensus Summit successfully identified key aspects of emergency surgery that need to be tackled to outline optimal strategy of care and definitive KPIs. Future work needs to expand on the work achieved here and in other forums, to define optimum care and robust, meaningful measurement tools of process and outcome. The WSES will lead the process in standardised KPI development. The summit acknowledged superb efforts to enhance emergency surgery care by others but felt an international collaboration and commitment was needed to implement and monitor these systems as soon as possible.

## References

[1] Tan BH, Mytton J, Al-Khyatt W, Aquina CT, Evison F, Fleming FJ, Griffiths E, Vohra RS (2017). A comparison of mortality following emergency laparotomy between populations from New York state and England. Annals Surgery.

[2] The Second Patient Report of the National Emergency Laparotomy Audit (NELA) December 2014 to November 2015 July 2016 http://www.nela.org.uk/reports accessed 23 Feb 2017.

[3] Santry HP, Madore JC, Collins CE, Ayturk MD, Velmahos GC, Britt LD (2015). Variations in implementation of acute care surgery: results from a national survey of university-affiliated hospitals. J Trauma Acute Care Surg.

[4] Tolstrup MB, Watt SK, Gögenur I (2016). Morbidity and mortality rates after emergency abdominal surgery: an analysis of 4346 patients scheduled for emergency laparotomy or laparoscopy. Langenbeck's Arch Surgery.

[5] Scott JW, Olufajo OA, Brat GA, Rose JA, Zogg CK, Haider AH, SalimA HJM (2016). Use of national burden to defineoperative emergency general surgery. JAMA Surg.

[6] Royal College of Surgeons in Ireland. Model of care for acute surgery: National Clinical Programme in Surgery [Internet]. RCSI; 2013. Available at http://www.rcsi.ie/files/surgery/20131216021838_Model%20of%20Care%20for%20Acute%20Surger.pdf. Accessed 12 Apr 2017.

[7] Hsee L, Devaud M, Middleberg L, Jones W, Civil I (2012). Acute surgical unit at Auckland City Hospital: a descriptive analysis. ANZ J Surg.

[8] Association of Surgeons of Great Britain and Ireland. Emergency general surgery: the future a consensus statement [Internet]. ASGBI. Available at http://www.asgbi.org.uk/consensus-statements/ accessed 12 Apr 2017.

[9] Sorelli PG, El-Masry NS, Dawson PM, Theodorou NA (2008). The dedicated emergency surgeon: towards consultant-based acute surgical admissions. Ann R Coll Surg Engl.

[10] Hameed SM, Brenneman FD, Ball CG, Pagliarello J, Razek T, Parry N (2010). General surgery 2.0: the emergence of acute care surgery in Canada. Can J Surg.

[11] Royal Australasian College of Surgeons. The case for the separation of elective and emergency surgery [Internet]. RACS; 2011. Available at http://www.surgeons.org/media/college-advocacy/submission-to-the-council-of-australian-government's-expert-panel-on-the-case-for-the-separation-of-elective-and-emergency-surgery/ accessed 12 Apr 2017.

[12] The Royal College of Surgeons of England. Separating emergency and elective surgical care: recommendations for practice [Internet]. RCSENG Professional Standards and Regulation; 2007. Available https://www.rcseng.ac.uk/library-and-publications/college-publications/year/accessed 12 Apr 2017.

[13] Professional Standards and Regulation Directorate: Royal College of Surgeons of England. Standards for Unscheduled Surgical Care: Guidance for providers, commissioners and service planners [Internet]. Publications Department, The Royal College of Surgeons of England; 2011. Available at: https://www.rcseng.ac.uk/library-and-publications/college-publications/year/accessed 12 Apr 2017.

[14] Resources for optimal care of emergency surgery Letterkenny 2016 978–0–09926109–9-9 Available http://dcra.ie/images/Resources_2016_Emergency_Surgery.pdf accessed 12 Apr 2017.

[15] Royal Australasian College of Surgeon (2015) Position paper of emergency surgery. Available at: https://www.surgeons.org/media/311630/2015-05-20_pos_fes-pst-050_emergency_surgery.pdf accessed 5 Oct 2017.

[16] General Surgeon Association of Australia (2010) 12-Point plan on emergency surgery. Available at: https://www.generalsurgeons.com.au/media/files/Publications/PLN%202010-09-19%20GSA%2012%20Point%20Plan.pdf. Accessed 5 Oct 2017.

[17] Ministry of Health New Zealand (2011) Targeting emergencies: shorter stays in emergency departments. Available at: https://www.health.govt.nz/search/results/Targeting%20emergencies%3A%20shorter%20stays%20in%20emergency%20department. Accessed 5 Oct 2017.

[18] Stewart B, Khanduri P, McCord C, Ohene-Yeboah M, Uranues S, Vega Rivera F, Mock C (2014). Global disease burden of conditions requiring emergency surgery. BJS.

[19] Tolstrup MB, Watt SK, Gögenur I (2017). Morbidity and mortality rates after emergency abdominal surgery: an analysis of 4346 patients scheduled for emergency laparotomy or laparoscopy. Langenbeck's Arch Surg.

[20] Scott JW, Olufajo OA, Brat GA, Rose JA, Zogg CK, Haider AH, Salim A, Havens JM (2016). Use of national burden to define operative emergency general surgery. JAMA surgery.

[21] Ogola GO, Haider A, Shafi S (2017). Hospitals with higher volumes of emergency general surgery patients achieve lower mortality rates: a case for establishing designated centers for emergency general surgery. J Trauma Acute Care Surg.

[22] Becher RD, Hoth JJ, Miller PR, Mowery NT, Chang MC, Meredith JW (2011). A critical assessment of outcomes in emergency versus nonemergency general surgery using the American College of Surgeons National Surgical Quality Improvement Program database. Am Surg.

[23] Ingraham A, Nathens A, Peitzman A, Bode A, Dorlac G, Dorlac W, Miller P, Sadeghi M, Wasserman DD, Bilimoria K (2017). Assessment of emergency general surgery care based on formally developed quality indicators. Surgery.

[24] Clavien PA, Puhan MA (2017). Measuring and achieving the best possible outcomes in surgery. Br J Surg.

[25] Scarborough JE, Schumacher J, Pappas TN, McCoy CC, Englum BR, Agarwal SK, Greenberg CC (2016). Which complications matter most? Prioritizing quality improvement in emergency general surgery. J Am Coll Surg.

[26] Nathan H, Dimick JB (2017). Quality accounting: understanding the impact of multiple surgical complications. Ann Surgery.

[27] Quiney N, Aggarwal G, Scott M, Dickinson M (2016). Survival after emergency general surgery: what can we learn from enhanced recovery programmes?. World J Surg.

